# Microelectrode Arrays for Detection of Neural Activity in Depressed Rats: Enhanced Theta Activity in the Basolateral Amygdala

**DOI:** 10.34133/cbsystems.0125

**Published:** 2024-06-05

**Authors:** Fanli Kong, Zhaojie Xu, Gucheng Yang, Qianli Jia, Fan Mo, Luyi Jing, Jinping Luo, Hongyan Jin, Xinxia Cai

**Affiliations:** ^1^ State Key Laboratory of Transducer Technology, Aerospace Information Research Institute, Chinese Academy of Sciences, Beijing 100190, China.; ^2^ School of Electronic, Electrical and Communication Engineering, University of Chinese Academy of Sciences, Beijing 100049, China.; ^3^ Obstetrics and Gynecology Department, Peking University First Hospital, Beijing 100034, China.

## Abstract

Depression is a common and severely debilitating neuropsychiatric disorder. Multiple studies indicate a strong correlation between the occurrence of immunological inflammation and the presence of depression. The basolateral amygdala (BLA) is crucial in the cognitive and physiological processing and control of emotion. However, due to the lack of detection tools, the neural activity of the BLA during depression is not well understood. In this study, a microelectrode array (MEA) based on the shape and anatomical location of the BLA in the brain was designed and manufactured. Rats were injected with lipopolysaccharide (LPS) for 7 consecutive days to induce depressive behavior. We used the MEA to detect neural activity in the BLA before modeling, during modeling, and after LPS administration on 7 consecutive days. The results showed that after LPS treatment, the spike firing of neurons in the BLA region of rats gradually became more intense, and the local field potential power also increased progressively. Further analysis revealed that after LPS administration, the spike firing of BLA neurons was predominantly in the theta rhythm, with obvious periodic firing characteristics appearing after the 7 d of LPS administration, and the relative power of the local field potential in the theta band also significantly increased. In summary, our results suggest that the enhanced activity of BLA neurons in the theta band is related to the depressive state of rats, providing valuable guidance for research into the neural mechanisms of depression.

## Introduction

Depression is a common neuropsychiatric condition that is indicated by symptoms like low mood, lack of interest or pleasure, and deterioration in sleep and appetite [1,2]. Globally, over 350 million people suffer from depression, and this number is still rapidly increasing, partly due to the COVID-19 pandemic [3]. In China, the lifetime prevalence of depressive disorders among adults is as high as 6.8%, yet only about 0.5% of these patients receive adequate treatment due to insufficient awareness of depression and a lack of medical resources [4]. Depression severely impacts patients’ social interactions and quality of life and imposes a heavy economic burden on healthcare systems worldwide [5]. However, the treatment of depression presents substantial challenges due to its complex etiology and unclear neural mechanisms [6,7].

Extensive research suggested that the activation of immune inflammation was highly associated with the formation and progression of depression [8–10]. Clinical studies have found that levels of inflammatory markers in the brain of depressed patients were abnormally elevated [11,12], and these elevations can usually be reversed by antidepressant medications [13,14]. Studies indicate that the inflammatory activation process disrupts neural circuits, making inflammatory patients more prone to mood disorders [15,16]. Lipopolysaccharide (LPS), a prevalent endotoxin, is present in the outer membrane of Gram-negative bacteria cell walls, which can cause a potent inflammatory reaction throughout the body by activating cytokine networks [17,18]. Many studies have found that repeated stimulation with LPS can lead to apparent depressive behaviors in animals, including reduced exploratory behavior and anhedonia [19–21]. Therefore, repeated LPS administration is used as a reliable model in depression research and is widely utilized for this purpose.

The amygdala is situated in the dorsomedial region of the temporal lobe of the brain [22], is a critical region for perceiving, distinguishing, and regulating emotions [23], and is thought to be associated with emotional disorders such as anxiety and depression [24]. The basolateral amygdala (BLA) is one of the 3 core subregions of the amygdala, primarily composed of excitatory projection neurons. It receives multimodal sensory information from areas like the thalamus and cortex [25,26], integrates these signals based on their value, and transmits them to target areas such as the hippocampus and the nucleus accumbens [27,28]. Prolonged research has shown a correlation between depression and abnormal activity in the BLA [29,30]. Research by Guo et al. [31] found that overexpression of SIRT1 in the neurons of the BLA induced depressive-like behaviors in normal mice, while specific knockdown of SIRT1 significantly reversed depressive symptoms in mice subjected to chronic unpredictable mild stress (CUMS). Zhang et al. [32] discovered that chronic restraint stress leads to dendritic hypertrophy in neurons of the BLA. Becker et al. [33] found that the neural pathway between the anterior cingulate cortex and the BLA becomes active when chronic pain triggers depressive-like behavior. Given the crucial role of the BLA in depression, detecting changes in the electrophysiological activity of the BLA during depression is of great importance for the treatment of the condition.

Microelectrode array (MEA), as a high-resolution, high-sensitivity, and multichannel detection tool, is widely used in neuroscience research [34,35]. For instance, Hou et al. [36] employed an ex vivo MEA to examine coronal brain slices of the medial prefrontal cortex (mPFC) in a rat model of circadian misalignment, revealing that circadian misalignment could impair synaptic plasticity in the mPFC and induce anxiety and depression-like behaviors in rats. Cao et al. [37] utilized implanted MEAs to investigate local field potential (LFP) activities in the mPFC and BLA of rats subjected to chronic unpredictable mild stress, finding that phase information transfer from the mPFC to the BLA was reduced during exploratory behavior in depressed rats. Additionally, Hu et al. [38] employed a 16-channel linear silicon probe to assess the impact of bisphenol A exposure on the central nervous system in juvenile mice, discovering that bisphenol A induced depression and social deficits in mice and led to hyperactivity in the amygdala neurons. Additionally, the application of diverse chemical materials to MEA devices can greatly expand the range of MEA applications [39]. Nanomaterials with excellent electrical properties can mitigate the high impedance caused by the reduced size of MEAs, enhancing the quality of neural signal recording. Certain conductive polymers possess good biocompatibility, which can improve the stability of MEAs in brain tissue, enabling the long-term stable acquisition of neural signals.

In this work, we designed a MEA tailored to the shape of the BLA and fabricated it via micro-electromechanical system thin-film deposition processes for long-term monitoring of neuroelectrophysiological signals in awake, freely moving rats. In order to improve the neural signal quality signals and detect the signals more sensitively, gold nanoparticles/ poly(3,4-ethylenedioxythiophene): poly(styrenesulfonate) (AuNPs/PEDOT:PSS) was employed to modify the MEA, significantly improving the sites impedance. We validated the reliability of LPS modeling through open-field tests (OFTs) and sucrose preference tests (SPTs) and recorded changes in electrophysiological signals before, during, and after continuous LPS administration for modeling. The study found that repeated LPS administration activated BLA neurons, significantly increasing delta power in the LFP and enhancing spike activity in the delta frequency band and pronounced periodic discharge characteristics emerged on the seventh day of LPS administration. Our BLA neuroelectrophysiological research provided crucial insights into the neural mechanisms of depression and may offer novel perspectives for its therapy.

## Materials and Methods

### Reagents and apparatus

3,4-Ethylenedioxythiophene (EDOT), gold (III) chloride (HAuCl_4_) trihydrate, and LPS from *Escherichia coli* were obtained from Sigma-Aldrich (Saint Louis, MO, USA). Poly(sodium-4styrenesulfonate) (PSS) was received from Herochem (Shanghai, China). phosphate-buffered saline (pH = 7.4) was received from Solarbio (Beijing, China). 1,1^′^-Dioctadecyl-3,3,3^′^,3^′^-tetramethylindocarbocyanine perchlorate (Dil) used for electrode staining was received from Acmec Biochemical Co., Ltd (Shanghai, China).

An electrochemical workstation used to perform electrochemical experiments was purchased from Gamry Instruments (Reference 600, PA, USA). The isoflurane anesthesia machine used to anesthetize rats during the electrode implantation surgery was obtained from RWD Life Science (R580S, Shenzhen, China). A Stereotactic apparatus for fixing the head of rats was purchased from Stoelting (model 51600, IL, USA). The electrode was implanted into the target brain region using a micropositioner obtained from David LOPF instrument (model 2662, CA, USA). A 128-channel neural data recording device (Blackrock, UT, USA) was utilized to acquire neural signals. The nanocomposites’ surface morphology was characterized using a scanning electron microscope (SEM) purchased from Hitachi (S-3500, Tokyo, Japan).

### Design and fabrication of MEA

**Fig. 1.**
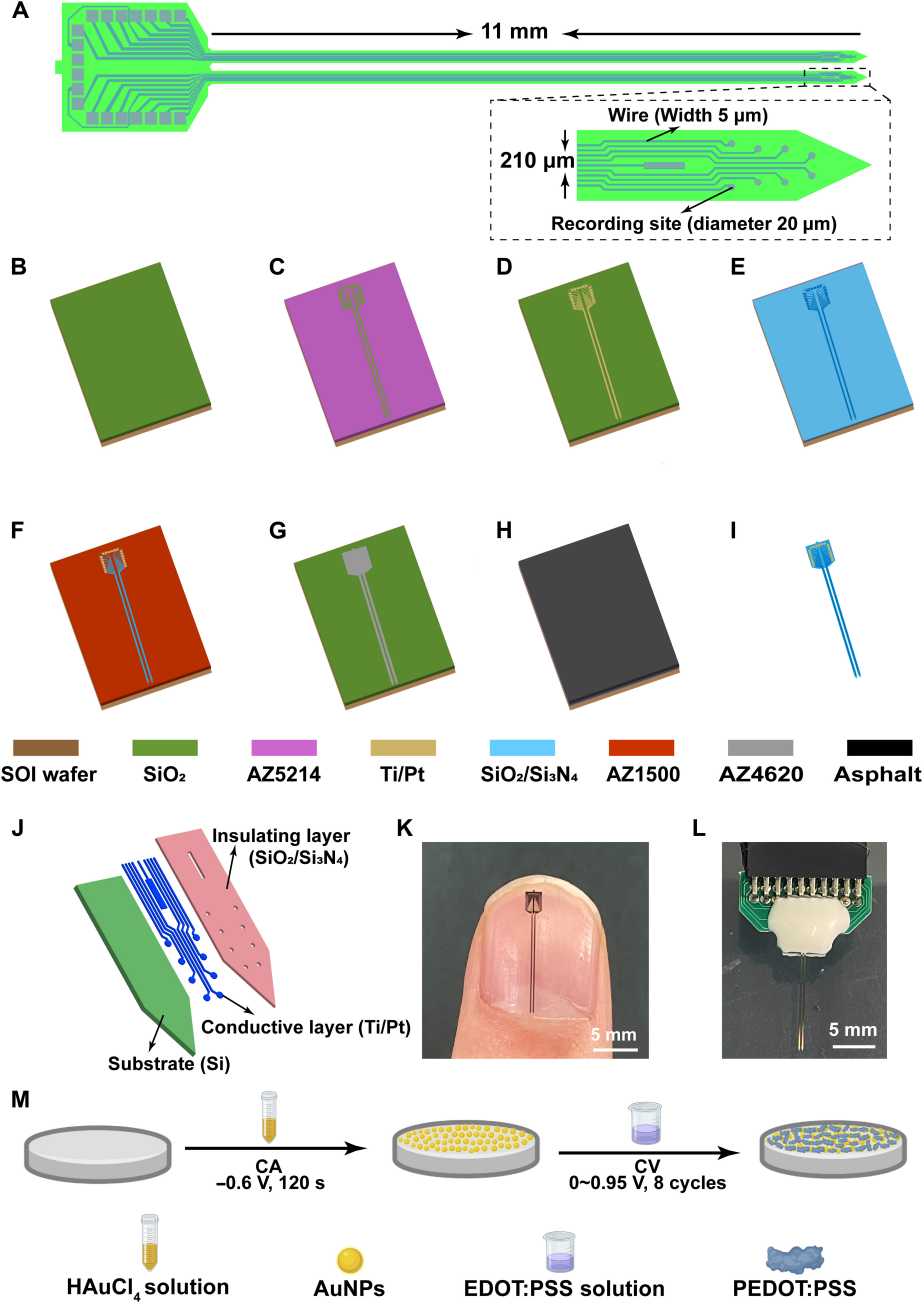
Schematic of MEA design and preparation. (A) Dual-shank electrode design, site arrangement, and dimensional parameters (inset: enlarged image of a shank tip). (B) SOI thermal oxidation. (C) The first photolithography and development to define the conductive layer of the MEA. (D) Sputtering of Ti/Pt and subsequent lift-off. (E) Forming an insulating layer through plasma-enhanced chemical vapor deposition. (F) Exposing the pads and sites of the MEA through photolithography and reactive ion etching. (G) Forming the shape of the MEA through photolithography and inductively coupled plasma reactive ion etching. (H) Applying asphalt on the front side of the SOI for protection. (I) Releasing the MEA through wet etching. (J) The fabricated electrode consists of a 3-layer structure. (K) MEA size compared to thumbnail. (L) MEA connected to back-end printed circuit board. (M) The sites of the MEA were progressively modified with AuNPs and PEDOT:PSS using electrochemical methods.

The schematic diagram of the MEA used for detecting neural signals in the BLA was shown in Fig. 1A. The BLA is located approximately 8 to 9 mm below the skull in rats. Considering individual variations among rats, the lengths of the 2 shanks of the MEA were designed to be 11 mm, allowing the MEA to be successfully implanted into the rat’s BLA. The recording sites of the MEA have a diameter of 20 μm, comparable to the size of mammalian neurons, enabling the detection of spikes from individual neural cells. The width of a shank is 210 μm, which is the minimum width that satisfies the requirement for the spacing between wires to be at least twice the width of the wire, thereby minimizing the size of the MEA to reduce implantation damage. Silicon is used as the substrate for the MEA due to its good biocompatibility, supporting in vivo monitoring for several weeks or more. Additionally, the silicon possesses excellent mechanical properties for precise implantation into deep brain regions.

MEAs were mass-produced on silicon-on-insulator (SOI, 25-μm device layer with 1-μm buried oxide layer and 25-μm handle layer) via micro-electromechanical system thin-film deposition process in a clean-room environment, and the entire fabrication process is shown in Fig. 1B to I. Initially, a 300-nm-thick SiO_2_ insulating layer was grown on the SOI surface through a thermal oxidation process (Fig. 1B). AZ 5214-E photoresist was then spin-coated, and the shape of the conductive layer was defined through photolithography and development processes (Fig. 1C). Subsequently, the Ti/Pt conductive layer (30 nm/250 nm) was formed through sputtering and lift-off techniques (Fig. 1D). To mitigate electrode bending caused by stress imbalance, 300 nm of SiO_2_ and 500 nm of Si_3_N_4_ were sequentially deposited as the electrode’s insulating layer using plasma-enhanced chemical vapor deposition (Fig. 1E). AZ1500 photoresist was then spin-coated on the SOI and used as a protective mask after photolithography. In the subsequent reactive ion etching process, the SiO_2_/Si_3_N_4_ layers above the electrode sites and electrode pads were etched away due to the lack of photoresist protection above, exposing the underlying conductive layer (Fig. 1F). Subsequently, similar to the previous process, AZ4620 photoresist was spin-coated, and a protective mask for the overall electrode structure was formed through photolithography and development. Then, the silicon adjacent to the electrodes was etched using inductively coupled plasma reactive ion etching until the buried oxide layer of the SOI was exposed, thereby shaping the MEA on the SOI (Fig. 1G). The front of the SOI was coated with asphalt to protect it, while the backside was etched with NaOH to release the electrodes (Fig. 1H and I). The completed MEA consists of a silicon substrate, an intermediate Ti/Pt conductive layer, and an upper SiO_2_/Si_3_N_4_ insulating layer (Fig. 1J). As illustrated in Fig. 1K, the fabricated MEA is smaller than a fingernail. For subsequent neural signal detection, we connected the electrodes to a backend printed circuit board using wire bonding and protected the lead sections with silicone (Fig. 1L).

### Modification of AuNPs/PEDOT:PSS nanocomposites

The impedance of unmodified bare electrodes can be as high as several megohms, posing a challenge in detecting neural signals within the range of hundreds of microvolts. Hence, we applied composite nanomaterials (AuNPs/PEDOT:PSS) composed of metal nanoparticles and conductive polymers to modify the MEA sites, with the aim of improving electrode performance and enhancing the MEA’s ability to detect weak signals. The modification process was depicted in Fig. 1M. Initially, in a 0.1% HAuCl_4_ solution, chronoamperometry with a −0.6-V voltage for 30 s was applied to deposit AuNPs onto the sites. Following that, a mixture of 0.1 M PSS and 20 mM EDOT was applied with cyclic voltammetry to deposit PEDOT:PSS onto the sites already modified with AuNPs. The cyclic voltammetry procedure was carried out within a voltage from 0 to 0.95 V with a scan rate of 100 mV/s, and a total of 8 cycles were completed. All electrochemical experiments were conducted using a 3-electrode system, with Pt acting as the counter electrode and Ag/AgCl (3 M KCl) as the reference electrode.

### Experimental design and drug administration

Adult male Sprague-Dawley rats weighing between 180 and 250 g were acquired from Beijing Vital River Laboratory Animal Technology Co., Ltd. These rats were kept in separate enclosures under controlled conditions in an environment with controlled temperature (25 ± 2 °C) and relative humidity (50% ± 5%), following a standard 12-h light/dark schedule (light off from 1200 to 2400). Except during the SPT, the rats had free access to water and food. All animal investigations were conducted in accordance with the guidelines set by the Beijing Association on Laboratory Animal Care and were authorized by Institutional Animal Care and Use Committee at the Aerospace Information Research Institute, Chinese Academy of Sciences (Approval number: AIRCAS-002).

**Fig. 2.**
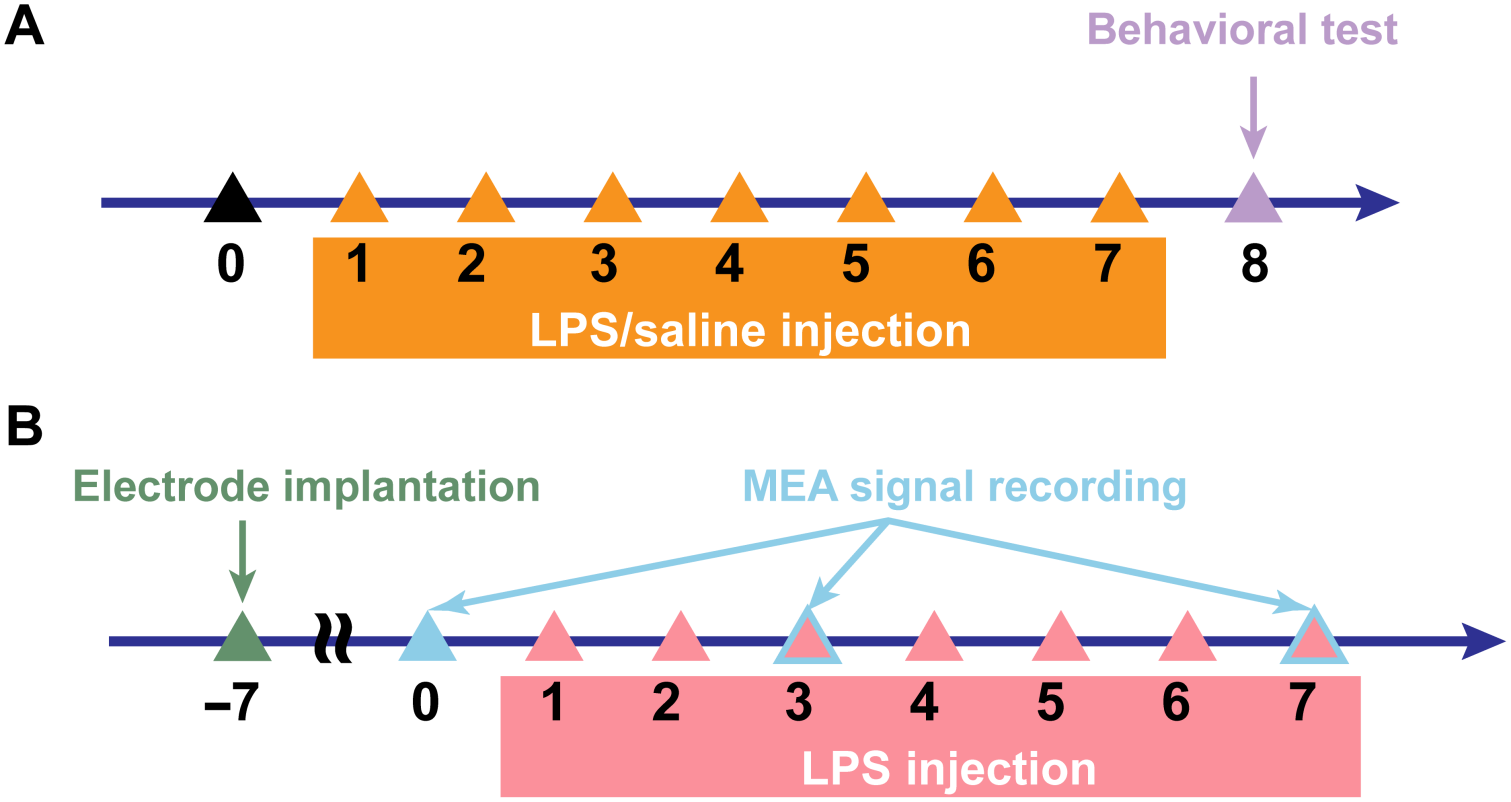
Experimental schedule. (A) Experimental schedule for behavioral test. Rats were injected with LPS or saline for 7 consecutive days, and behavioral experiments were performed 24 h after the last injection. (B) Experimental schedule for electrophysiologic recording. MEA was implanted into the BLA about 7 d before modeling, and electrophysiological signals were recorded from the BLA before LPS administration and 5 h after administration on the third and seventh days.

The LPS administration was as consistent with previous earlier studies [40,41]. LPS was dissolved in sterile saline and supplied at a dosage of 0.5 mg/kg for a duration of 7 consecutive days. To verify the effects post-LPS administration, we conducted an OFT (*n* = 6) and an SPT (*n* = 6) 24 h after the last LPS administration. Additionally, we used saline in place of LPS as a control group for the behavioral experiments (*n* = 5), the experimental procedure for behavioral test was as outlined in Fig. 2A.

For the electrophysiological signal detection group (*n* = 5), the experimental procedure was as outlined in Fig. 2B. The MEA needed to be implanted into the rat’s BLA. Prior to surgery, the surgical table and instruments were sterilized with 75% alcohol. The rat was anesthetized with isoflurane (5% for induction, followed by 1% to 2% to maintain anesthesia). The rat head was stably secured in a stereotaxic frame and carefully adjusted to ensure the skull was horizontal. The rat’s cranial head hair was trimmed with clippers, and the scalp was incised with scissors to reveal the cranium. Following a craniotomy, the MEA was carefully implanted into the rat’s BLA at certain coordinates (anterior-posterior: −2.7 mm, medial-lateral: 4.8 mm, dorsal-ventral: −7.9 mm) using a micropositioner at a slow speed of 10 μm/s. Once the electrode reached the target location, the MEA was then secured to the rat’s cranium utilizing dental cement. After the surgery, the rat was allowed to rest for 7 d to return to normal condition before proceeding with further experiments. We first recorded the normal electrophysiological activity of rat BLA neurons 1 d before starting the modeling, followed by a continuous 7-d regimen of LPS injections. We then recorded the neuronal electrophysiological activity again 5 h after the LPS injection on both the third and seventh days and compared these data with the previously obtained data for analysis.

### Behavioral tests

#### Open-field test

The OFT was employed to assess the locomotor activity and exploratory behavior of rats, with the experimental procedure as described in related studies [42]. OFT was conducted in a dimly lit testing room between 1200 and 1700. Rats were brought into the behavioral room 30 min before the test to acclimatize to the environment. The open field consisted of a black square cage with a size of 100 cm × 100 cm × 40 cm, above the arena equipped with a camera to record the rat’s movements in real time. Initially, the rat was positioned in the middle of the arena for the experiment, and after a 30-s adaptation period, its spontaneous activities were recorded for 5 min. Prior to each trial, the arena was meticulously sanitized with alcohol to eradicate any scents. The locomotion of the rats was retrospectively analyzed offline using EthoVision XT video tracking software (Noldus, Wageningen, Netherlands).

#### Sucrose preference test

According to relevant studies [43], the procedure for the SPT was as follows. The 72 h before the official start of the experiment served as the adaptation phase: 2 bottles containing a sucrose with a concentration of 1% (W/V) were initially inserted in each cage. After a period of 24 h, one of the sucrose solutions was substituted with water. After a 24-h period of withholding food and water, 2 bottles of solution were weighed and inserted in the cage, one containing water and the other containing the sucrose solution. The placements of the water and sucrose were randomized and alternately interchanged every 30 min. One hour later, the bottles were removed and weighed to calculate their consumption. Sucrose preference was quantified as the proportion of the consumption of sucrose solution consumed relative to the combined intake of both the sucrose solution and water.

### Histology

Histology was used to verify the accurate implantation of the MEA into the BLA. Before implantation, each shank of the MEA was coated with Dil. Under deep anesthesia, the rats underwent surgery to incise the thorax and reveal the heart. The heart was rapidly perfused with 0.9% saline to flush out the blood from the body, followed by slow perfusion with 4% paraformaldehyde solution to stabilize the brain tissue. Subsequently, the rat’s brain was removed and subjected to dehydration using solutions containing 20% and 30% sucrose. The brain was sectioned into 40-mm slices utilizing a cryostat (CM1950, Leica Biosystems, Wetzlar, Germany), and the electrode implantation sites were examined employing a microscope based on the rat brain atlas [44]. As shown in Fig. S1, the electrodes were precisely implanted into the BLA.

### Data acquisition and analysis

Neural signal collection was conducted while the animals were awake and free to move. The MEA was connected to an electrophysiological recording system via a 10× preamplifier, capturing neural signals at a sampling rate of 20 kHz. The raw neural signals can be extracted as spike signals by a high-pass filter (>200 Hz) and LFP signals by a low-pass filter (<200 Hz). The spike extraction threshold was set at 3 times the noise baseline. Spike and LFP signals were analyzed using NeuroExplore software (Nex Technologies, CO, USA). Graphing and statistical analysis were performed using Origin 2018 (OriginLab Corporation, MA, USA). All data are reported as means ± standard error (SE). Analysis of variance (ANOVA) with Tukey’s post hoc test was employed to analyze significance. A *P* value below 0.05 was deemed to be statistically significant.

## Results

### Morphology and characterization of MEA

**Fig. 3.**
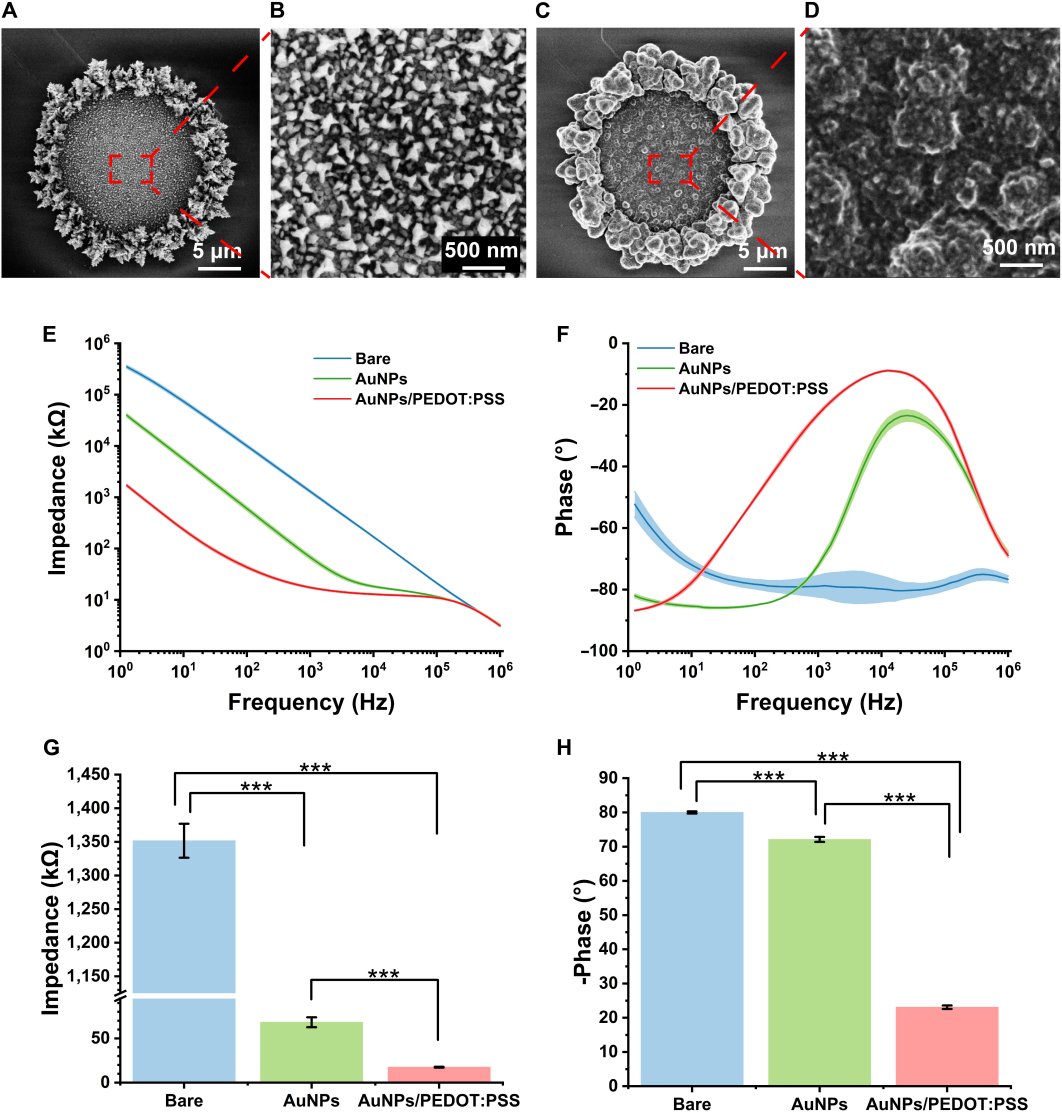
SEM and EIS characterization of electrode nanocomposites. (A) SEM images of sites modified with AuNPs at 3kx magnifications. (B) SEM images of sites modified with AuNPs at 30kx magnifications. (C) SEM images of sites modified with AuNPs/PEDOT:PSS at 3.5kx magnifications. (D) SEM images of sites modified with AuNPs/PEDOT:PSS at 40kx magnifications. (E) The impedance variation with frequency from 1 to 10^6^ Hz for different modified interfaces. (F) The phase variation with frequency from 1 to 10^6^ Hz for different modified interfaces. (G) Comparison of impedance at 1 kHz for different modified interfaces. (H) Comparison of phase delay at 1 kHz for different modified interfaces. AuNPs/PEDOT:PSS modification significantly improves the impedance and phase delay of MEA. Data are presented as means ± SE. ***P < 0.001 (one-way ANOVA with Tukey's post hoc test).

In this work, the surfaces of MEA sites were modified with AuNPs/PEDOT:PSS to enhance the electrodes’ responsiveness to weak signals, as unmodified bare electrodes have high impedance, making it difficult to detect electrophysiological signals directly. SEM is a fundamental and crucial method for characterizing material morphology, and we used it to observe the changes in morphology during the site modification process. The surface morphologies of bare electrode and AuNPs- and AuNPs/PEDOT:PSS-modified electrodes were shown in Fig. S2A and B, and Fig. 3A to D, respectively. Compared to the smooth surface of bare electrodes, it was observed that the deposition of AuNPs on the sites resulted in a rough surface formed by angular pebble-like accumulations. During the deposition of PEDOT:PSS, it accumulated layer by layer on the surface of AuNPs, gradually filling the gaps between the AuNPs and ultimately creating a surface morphology resembling a pile of small spheres. The unique surface structure of AuNPs/PEDOT:PSS has the ability to augment the electrode’s specific surface area, facilitating the adhesion of neural cells and enhancing its sensitivity for detection. The energy-dispersive x-ray spectroscopy results for AuNPs- and AuNPs/PEDOT:PSS-modified electrodes were provided in Fig. S2C and D. The Au element observed in Fig. S2C originates from the AuNPs, while the S element observed in Fig. S2D comes from PEDOT:PSS.

Electrochemical impedance spectroscopy (EIS) is an extremely valuable tool that can provide detailed electrical information about an interface, which is beneficial for the selection and optimization of interfacial materials to fulfill the demands of diverse applications. EIS measurements were conducted in a phosphate-buffered saline solution with a frequency range from 10^6^ to 1 Hz and a sinusoidal voltage perturbation of 5 mV. The impedance and phase changes with frequency for bare electrodes, AuNPs-modified electrodes, and AuNPs/PEDOT:PSS-modified electrodes were depicted in Fig. 3E and F. As 1 kHz is the fundamental frequency of action potentials from neurons surrounding the implanted electrodes, the impedance and phase at the 1-kHz frequency for different modified interfaces were compared (Table S1 and Fig. 3G and H). The mean impedance of the AuNPs/PEDOT:PSS-modified sites was 17.44 kΩ. This represents a decrease of more than 98% compared to the bare electrodes (*P* < 0.001) and is nearly one-fourth of the impedance seen in the AuNPs-modified sites (*P* < 0.001) (). Regarding phase delay, the bare electrode sites exhibited an average phase delay of −79.0°, while it was −72.1° after AuNPs modification and −23.1° for AuNPs/PEDOT:PSS-modified sites (Fig. 3H). Through the investigation of impedance and phase delay, we have determined that MEAs enhanced with AuNPs/PEDOT:PSS demonstrate exceptional electrical capabilities. This improvement can strengthen our ability to identify electrophysiological signals with greater sensitivity. The MEAs used in this study are all from the same SOI, and there is little difference in the electrical properties of different MEAs (Fig. S3).

### Effects of LPS treatment in OFT and SPT

**Fig. 4.**
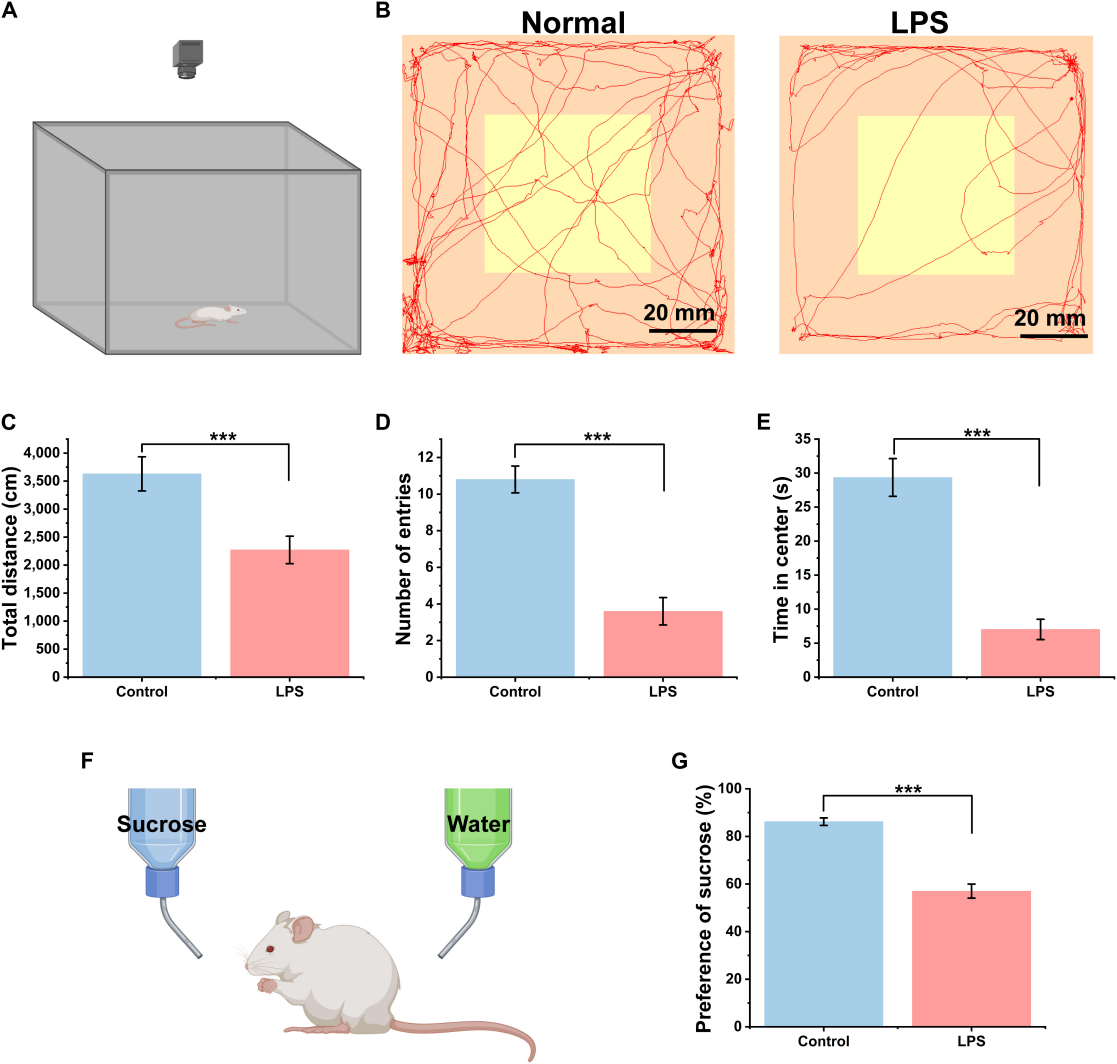
The impact of LPS administration on depression-like behaviors. (A) The schematic diagram of the OFT. (B) The movement trajectories of rats in the open field. Left: Control group. Right: LPS administration group. The yellow square represents the central area of the open field. (C to E) The impact of LPS administration on rats in the OFT was assessed by measuring the total movement distance (C), the number of entries into the central area (D), and the time spent in the central area (E). LPS treatment significantly reduced the rats' locomotor and exploratory behavior in OFT. (F) The schematic diagram of the SPT. (G) The effect of LPS administration on the preference sucrose in rats. LPS administration reduced the sucrose preference in rats. Data are presented as means ± SE. ***P < 0.001 (one-way ANOVA with Tukey's post hoc test).

Twenty-four hours after the final LPS injection, the effects of LPS on rat behavior were observed through the OFT and SPT. The OFT assesses locomotor activity and anxiety-like behavior (Fig. 4A). We defined a central area in the middle of the arena, which occupied one-fourth of the total arena. The demarcation of the center was established within the video analysis software, and there were no boundary markings in the arena itself, so the rats’ movement was not influenced by any such markings. The locomotor tracks of rats in the arena for the LPS administrated group and control group were shown in Fig. 4B and C, respectively. Control group rats actively walked and explored around the area, whereas rats administered with LPS tended to walk along the walls of the arena or stay in the corners, and they less frequently ventured into the center. We quantified the total movement distance, the number of entries into the central area, and the duration of time spent in the central area between the 2 groups. As shown in Fig. 4C to E, all 3 metrics significantly decreased (*P* < 0.001) after LPS administration. These results indicated that LPS treatment significantly reduced the rats’ locomotor and exploratory behavior. The SPT was utilized to evaluate anhedonia, which is one of the core indications of depression (Fig. 4F). As shown in Fig. 4G, normal rats have a high preference for sucrose solution. However, a significant reduction was observed after LPS treatment (*P* < 0.001). Overall, consistent with previous reports, repeated LPS administration increased anxiety-like behavior in rats and decreased their ability to experience pleasure, all of which indicate that the rats entered a state of depression.

### LPS administration resulted in enhanced neuronal activity in the BLA

**Fig. 5.**
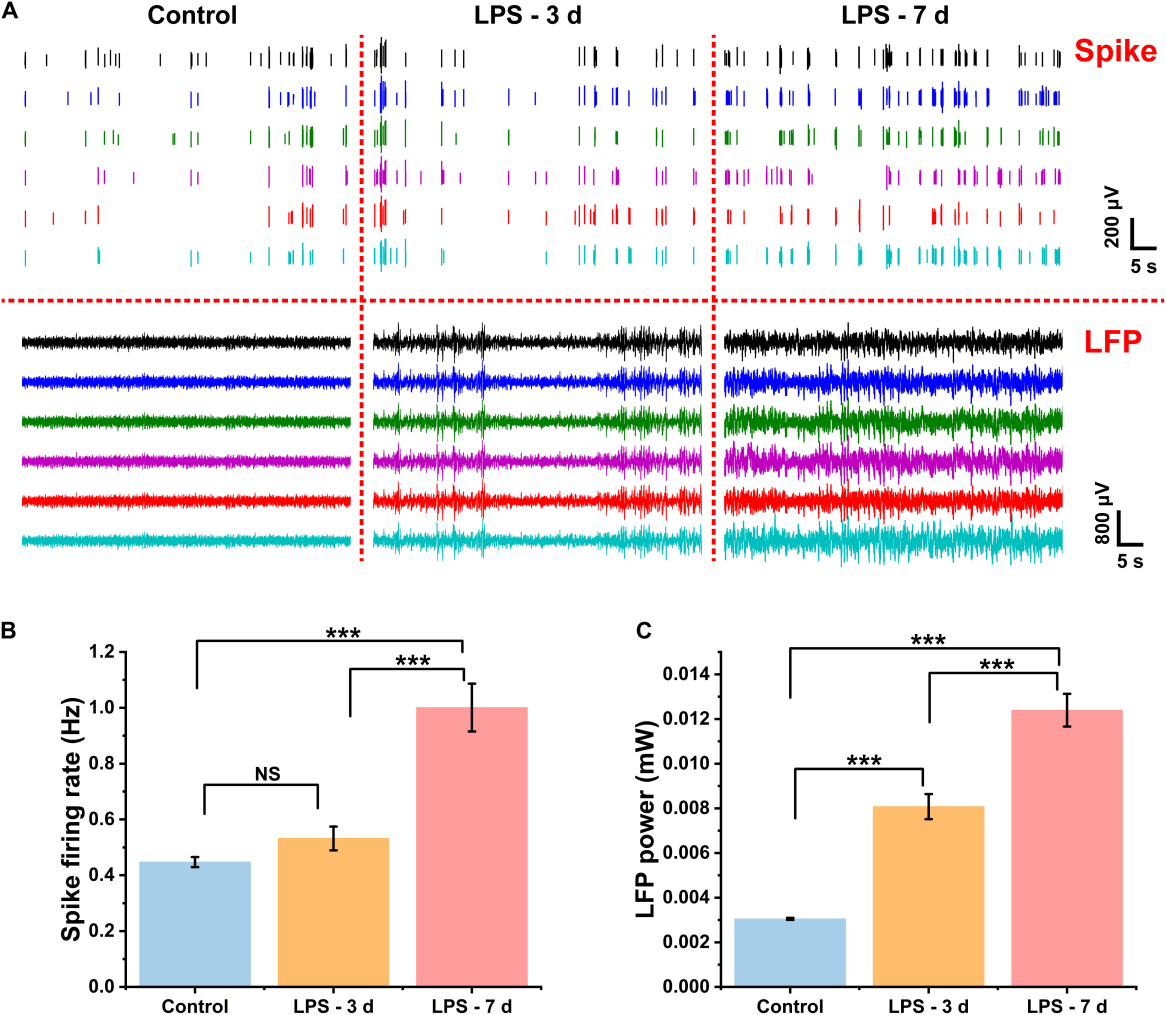
Effects of LPS administration on BLA neuronal activity. (A) Electrophysiological signals in 6 typical channels detected by MEA in BLA. Top: Spike. Bottom: LFP. (B) Changes in spike firing rate before, during, and after LPS administration. Spike firing rate increased progressively with LPS administration. (C) Changes in LFP power before, during, and after LPS administration. LFP power gradually increased with LPS administration. Data are presented as means ± SE. ***P < 0.001 (one-way ANOVA with Tukey's post hoc test). NS, not signigicant.

To understand the intrinsic neural mechanisms behind the depressive state in rats after LPS administration, we implanted a MEA into the rats’ BLA and recorded changes in neurophysiological data before, during, and after LPS administration. Neurons in the BLA were closely adhered to the sites at the tip of the MEA, allowing for the simultaneous detection of LFPs and spike signals. LFP represent the sum of extracellular low-frequency electrophysiological signals in the neural tissue around the recording sites, broadly reflecting the changes in the electrical field caused by the collective activity of neuronal populations near the recording site. Spikes, which are often referred to as action potentials, and their various firing patterns are essential for the transmission and processing of information in the neuronal system of the brain. Therefore, analyzing spikes and LFPs can provide insights into the neural activity characteristics of depression. Fig. 5A showed real-time recordings of neural spikes and LFPs across 6 different channels over a 60-s period. Under normal conditions, the BLA exhibited sporadic and sparse spike firing, with LFP fluctuations being relatively weak, mostly ranging between −96 and 94 μV. On the third day of LPS administration, spike discharges increased compared to before administration, and LFP fluctuations became more intense, with amplitudes mostly ranging between −128 and 134 μV. By the seventh day of LPS administration, both spike discharges and LFP fluctuations further intensified, with LFP amplitudes concentrating between −183 and 178 μV.

We characterized neuronal activity using spike firing rates and LFP power (in 1 to 30 Hz), with results shown in Fig. 5B and C. Before LPS administration, the average spike firing rate was 0.45 Hz. On the third day of administration, the spike firing rate slightly increased to 0.53 Hz (*P* = 0.58). By the seventh day, it had significantly risen to 1 Hz (*P* < 0.001), more than doubling compared to before administration. In terms of LFP power, it was 3.05 mW on the first day, significantly increased to 8.08 mW on the third day (*P* < 0.001), and continued to rise to 12.4 mW by the seventh day (*P* < 0.001). These results indicate that more neurons in the BLA were activated after LPS administration.

### Abnormal theta activity in spikes and LFPs after LPS administration

**Fig. 6.**
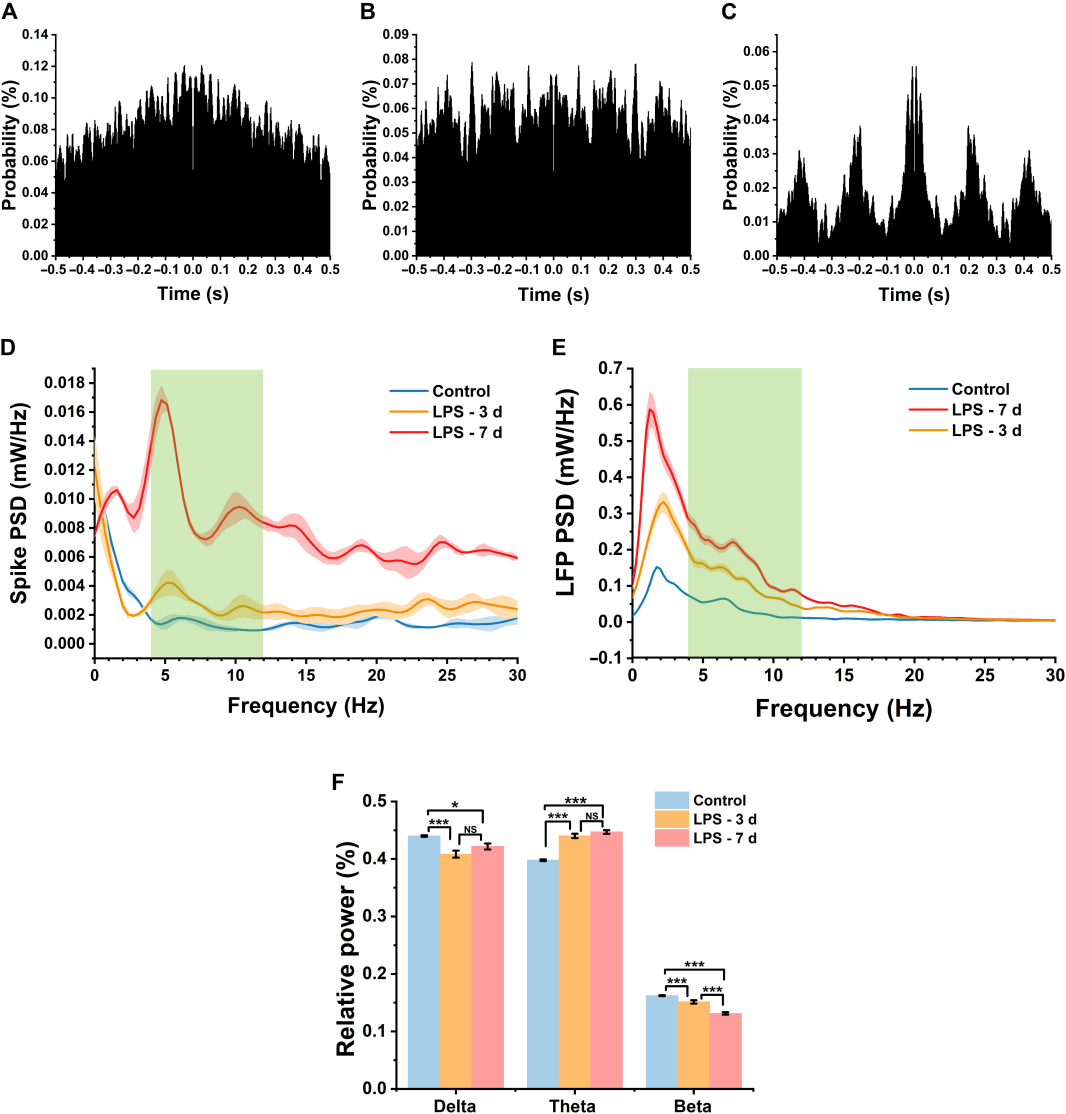
LPS administration resulted in enhanced neuronal theta activity in the BLA. (A) Spike autocorrelation analysis of typical channels before LPS administration. (B) Spike autocorrelation analysis of typical channels on the third day of continuous LPS administration. (C) Spike autocorrelation analysis of typical channels on the seventh day of continuous LPS administration. With the administration of LPS, spike showed obvious periodic oscillations. (D) Variation of spike PSD within 0 to 30 Hz. The green part is 4 to 12 Hz. As LPS was administered, the characteristic peaks in the theta band became more pronounced. (E) Variation of LFP PSD within 0 to 30 Hz. The green part is 4 to 12 Hz. The PSD curves of different periods show characteristic peaks in the theta band. (F) Changes in the relative power of LFPs in different frequency bands. The relative power of the LFP in theta band rises gradually with LPS administration. Data are presented as means ± SE. *P < 0.05, ***P < 0.001 (one-way ANOVA with Tukey's post hoc test).

Autocorrelation of spikes within a single channel was examined, and the resulting autocorrelation histograms before, during, and after LPS administration modeling were shown in Fig. 6A to C. Compared to the random and unpatterned discharges before administration, the autocorrelation histogram envelope on the third day of administration became similar to a cosine function, showing less distinct periodic oscillations. On the seventh day, the periodic firing characteristics became more pronounced, with a firing cycle of 5 Hz. Consequently, we further analyzed and obtained the power spectral density (PSD) curves of spikes within the 0- to 30-Hz range. We divided the 1- to 30-Hz range into 3 bands: delta (1 to 4 Hz), theta (4 to 12 Hz), and beta (12 to 30 Hz), with a focus on the theta band where 5 Hz is located. As shown in Fig. 6D, the PSD was generally flat before administration, with a small characteristic peak in the theta band (around 6 Hz). On the third day of LPS administration, although the overall PSD curve remained flat, a more pronounced characteristic peak appeared in the theta band (around 5 and 10 Hz). On the seventh day, the characteristic peak values in the theta band of the PSD further increased from the third day. Since spike firing became faster after administration, the PSD curves after administration were also overall higher than those before administration.

As a reflection to the collective activity of neurons, the PSD of LFPs was also analyzed, with results shown in Fig. 6E. Although the PSD curves of the 3 groups of LFPs differ in amplitude due to variations in LFP power, they share similarities: the power was mainly concentrated in the 0- to 20-Hz frequency range, with characteristic peaks within the 1- to 2-Hz and 6.5- to 7.5-Hz ranges. We analyzed changes in the relative power of LFPs across 3 different frequency ranges following LPS administration, as shown in Fig. 6F and Table S2. After LPS administration, the power proportions of both the delta and beta ranges decreased, while the power proportion of the theta range showed a steady increase. We found that the delta band dominated before LPS administration, but after LPS administration, the dominant frequency band shifted from delta to theta. The simultaneous increase in activity in the theta band for both spikes and LFPs suggests that theta activity in the BLA could serve as a biomarker for depression.

## Discussion

Precise detection of brain electrophysiological activity is a crucial method for understanding the micromechanisms of neuro-related diseases. In vivo electrophysiological measurements in awake and freely moving animals offer a closer approximation to the animal’s natural state compared to brain slices and anesthetized conditions, thus providing an advantage in exploring the neural mechanisms of diseases. MEA is an important tool for detecting neural electrophysiological activity, offering advantages such as high spatiotemporal resolution, versatility, and customizability. MEA is minimally invasive when implanted into target brain areas and can detect the LFP representing group neuron activity and single-cell level spike firings in real time without causing major cells damage. Nanomaterials, due to their unique structures and properties, have been widely applied in MEA site modification, becoming a crucial means to improve electrode performance. In our study, we used AuNPs/PEDOT:PSS for site modification, combining the advantages of metallic nanomaterials and conductive polymers with lower impedance and smaller phase delay compared to AuNPs-modified sites.

Numerous studies in both humans and rodents have demonstrated that the activation of peripheral and central immune cells plays an important role in neuropsychiatric disorders like anxiety and depression [45]. Elevated expression of proinflammatory cytokines has been observed in the blood as well as brain tissues of many depressed patients and model animals [9]. In our study, we administered high doses of LPS to rats for 7 consecutive days. LPS challenge has been widely proven to induce immune activation in various brain regions, leading to severe neuroinflammation. We assessed the rats’ anxiety and depressive states using 2 common behavioral paradigms: the OFT and the SPT. The results indicated that LPS challenge resulted in reduced movement distance and fewer entries into the central area in OFT, as well as a decreased preference for sucrose solution in SPT. These behavioral changes were evidence of depressive-like symptoms in rats.

BLA is a vital brain region associated with emotions, receiving projections from areas such as the cortex and hippocampus, integrating and transmitting information to downstream brain regions [46]. The BLA is essential for the manifestation and control of emotions, and abnormal neuronal activity in the BLA is linked to the emergence of diverse psychological conditions, such as anxiety and depression [29,30]. In our study, we used a LPS-induced model of depression and employed MEAs to investigate the neural activity in the BLA. Our study confirmed that overactivity in the BLA is associated with the development of depression, suggesting that modulating BLA neural activity could be an effective method for alleviating depression. These findings indicated that BLA neurons are extensively activated and exhibit frequent firing during depression. Our study confirmed that overactivity in the BLA is associated with the development of depression, suggesting that modulating BLA neural activity could be an effective method for alleviating depression.

LFPs reflect the activity of neuronal populations, and their oscillations across different rhythms are indicative of various network dynamics and information encoding [47]. We divided LFP oscillations into 3 different frequency bands. The analysis indicates that compared to before LPS injection, the relative power of both delta and beta frequency bands decreased after LPS administration, while the relative power of the theta frequency band significantly increased. Additionally, there was a shift in the dominant frequency band from delta to theta. In terms of analysis of spikes, we found that the characteristic peaks in the theta band of the spike PSD curve became more pronounced after LPS administration, suggesting a certain association between neuronal firing and the theta rhythm. The autocorrelation analysis of spikes also confirmed this. Similar findings regarding LFPs have also been observed in other studies related to depression, where theta activity was considered to be associated with the expression of emotion [48,49]. Given the correlation between spikes and LFPs, we hypothesize that the enhanced rhythmicity of spike firing in the theta band is one of the reasons for the enhancement of power in the theta band in the LFP. This insight underscores the potential relevance of BLA theta activity alterations in the pathophysiology of depression, suggesting that addressing these changes could be a strategic method in treating depression.

The mPFC is an upstream brain region of the BLA, sending mPFC in the expression of anxiety and depression in rodents [50,51]. The mPFC communicates remotely with its downstream brain regions mainly through theta oscillations, and a number of studies have found that depression is associated with abnormal theta communication in the mPFC-BLA [52,53]. Thus, the enhanced theta activity of BLA during LPS administration found in this study could be a result of mPFC regulation. Neurotransmitters represent another form of communication between neurons, with the BLA primarily consisting of glutamatergic neurons [54]. Investigating how glutamate concentration changes in the BLA after LPS administration and correlating these with electrophysiological signals can reveal more characteristics of depressive neural activity. The MEA used in this study can be customized according to experimental requirements, and the sites, once modified with specific nanomaterials and enzymes, can facilitate in vivo, in situ real-time detection of glutamate [55]. Therefore, the next phase of our research will focus on the synchronous detection of in vivo electrophysiological signals and neurotransmitter signals in the mPFC and BLA, aiming to provide further neurobiological insights into the connection between inflammation and depression.

In summary, in this work, a dual-shank MEA for detecting neurophysiological information in the BLA of rats with LPS-induced depression was designed and fabricated. We modified the MEA sites with AuNPs/PEDOT:PSS, making the electrode impedance at 1 kHz decreased from 1.35 MΩ to 17.44 kΩ. Our results indicated that neuronal activity in the BLA increased after LPS administration, with both LFP power and spike firing rate showing an uptick. Notably, the most significant change in LFP power occurred in the theta frequency band. Analysis of spikes revealed that after LPS administration spikes tended to fire in the theta rhythm, with periodic oscillations around 5 Hz, suggesting that this abnormal spike discharge may be related to the neural mechanisms of depression. Our work provided tools for deep brain nuclei studies in depression and offers insights into the relationship between inflammation and depression through our investigation of neuronal activity in the BLA.

## Data Availability

All data needed to support the conclusions in the paper are provided in the paper and the Supplementary Materials.
