# Supplementary material for: Microelectrode Arrays for Detection of Neural Activity in Depressed Rats: Enhanced Theta Activity in the Basolateral Amygdala
Source: Cyborg Bionic Syst. 2024 Jun 5;5:0125. doi: 10.34133/cbsystems.0125 (PMC11151173; doi:10.34133/cbsystems.0125)
Supplement: Supplementary 1 — Figs. S1 to S3 Tables S1 and S2 [file cbsystems.0125.f1.docx]

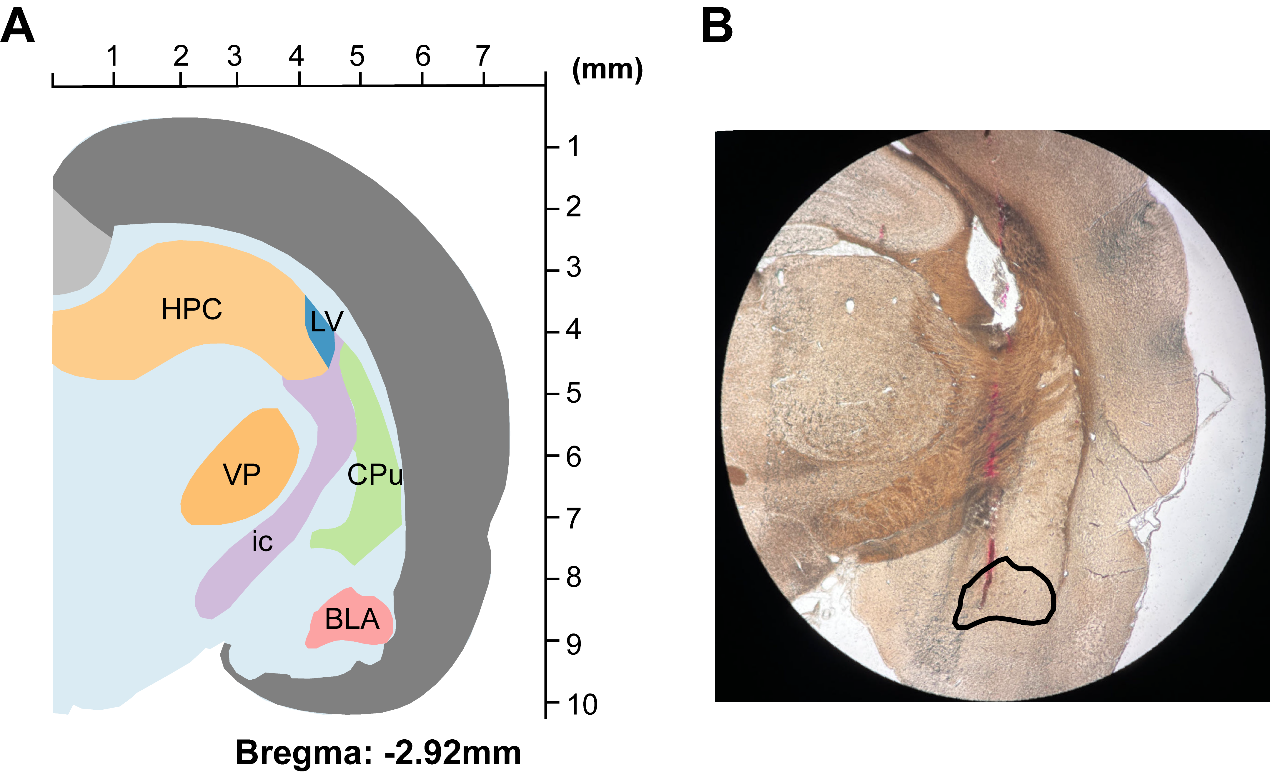


**Fig. S1.** Localization of MEA in brain regions. (A) Schematic diagram of rat brain atlas. (B) Coronal section of rat brain showing the location of MEA. The red mark is the location of MEA implantation.


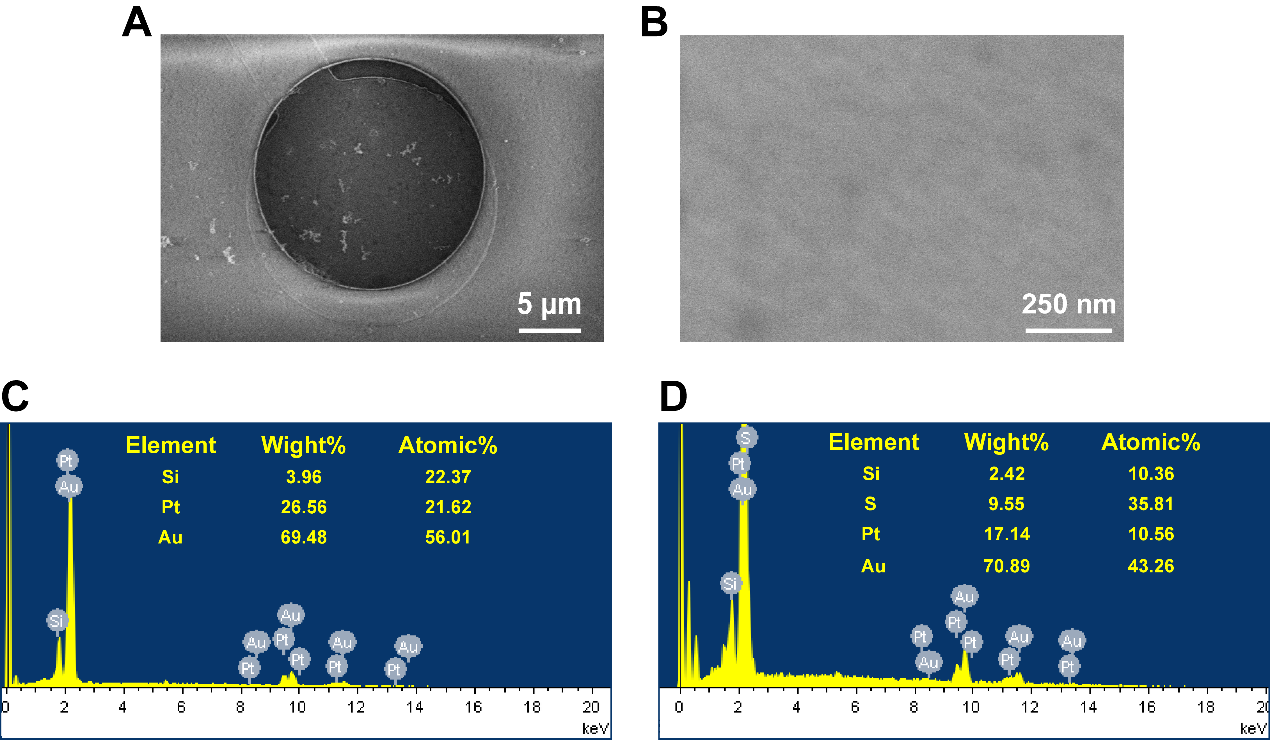


**Fig. S2.** MEA modification characterization. (A) SEM images of bare electrodes at 3.5kx magnifications. (B) Scanning electron microscope images of bare electrodes at 100kx magnifications. (C) The EDS of the AuNPs modified electrode. (D) The EDS of the AuNPs/PEDOT:PSS modified electrode.


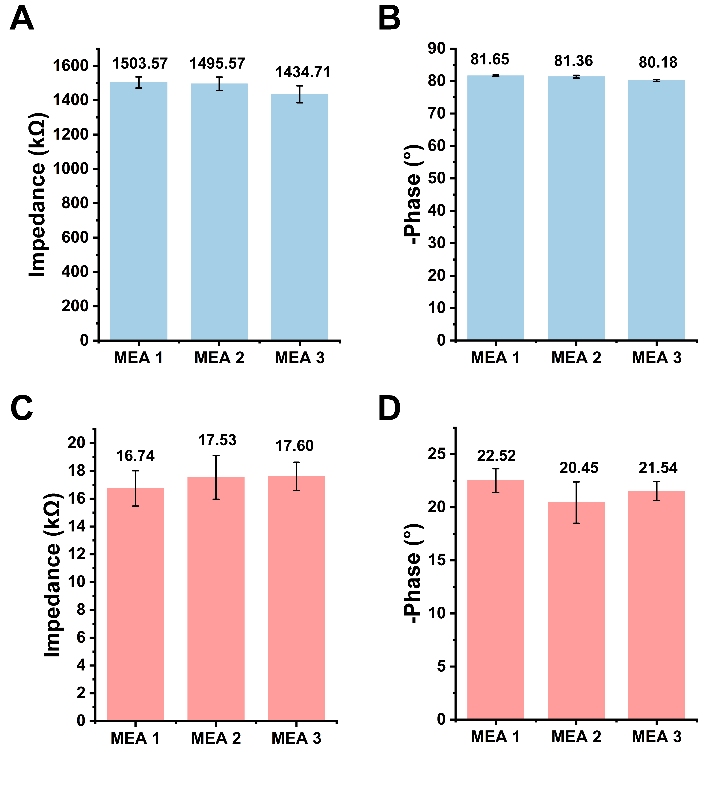


**Fig. S3.** Electrical performance characterization of different MEAs in the same batch. (A) Difference in impedance of bare electrodes at 1 KHz. MEA 1: 1503.57 ± 32.56 kΩ, MEA 2: 1495.57±38.88 kΩ, MEA 3: 1434.71 ± 349.65 kΩ. (B) Phase difference of bare electrodes at 1 KHz. MEA 1: 81.65 ± 0.23°, MEA 2: 81.36±0.42°, MEA 3: 80.18±0.30°. (C) AuNPs/PEDOT:PSS modified electrode impedance difference at 1 KHz. MEA 1: 16.74 ± 1.27 kΩ, MEA 2: 17.53 ± 1.58 kΩ, MEA 3: 17.60 ± 1.02 kΩ. (D) AuNPs/PEDOT:PSS modified electrode phase difference at 1 KHz. MEA 1: 22.52 ± 1.12°, MEA 2: 20.45 ± 1.95°, MEA 3: 21.54 ± 0.89°. Data are presented as mean ± SE.

**Table S1. Average impedance and phase of different nanomaterial modified electrodes at 1 kH.**

| Modifying material | Average impedance (kΩ) | Average phase delay (°) |
| --- | --- | --- |
| Bare | 1351.67 ± 25.24 | 80.0 ± 0.3 |
| AuNPs | 68.20 ± 5.62 | 72.1 ± 0.7 |
| AuNPs/PEDOT:PSS | 17.44 ± 0.48 | 23.1 ± 0.5 |

**Table S2. Relative power of LPF in each frequency band during LPS administration modelling.**

| Band (Hz) | Control (%) | LPS - 3 d (%) | LPS - 7 d (%) |
| --- | --- | --- | --- |
| Delta | 43.99± 0.13 | 40.84 ± 0.61 | 42.18 ± 0.53 |
| Theta | 39.79 ± 0.14 | 44.02 ± 0.38 | 44.69 ± 0.32 |
| Beta | 16.22 ± 0.09 | 15.14 ± 0.29 | 13.13 ± 0.21 |
